# Impacts of and adaptation to climate change on the oil palm in Malaysia: a systematic review

**DOI:** 10.1007/s11356-021-15890-3

**Published:** 2021-08-16

**Authors:** Ahmed Abubakar, Mohd Yusoff Ishak, Abdullah Ahmad Makmom

**Affiliations:** Faculty of Forestry and Environment, Universiti Putra Malaysia, UPM, 43400 Serdang Selangor, Malaysia

**Keywords:** Extreme event, Temperature, Rainfall, Variability, Oil palm, Malaysia

## Abstract

The interaction and the interplay of climate change with oil palm production in the Southeast Asia region are of serious concern. This particularly applies in Malaysia due to its rank as the second largest palm oil producer in the world. The anthropogenic activities and the agroecological practices in oil palm plantation, including excessive use of fertilisers, bush fire due to land clearing, and cultivation on peatland, have exacerbated the effects of climate change featuring extreme events, drought, flooding, heatwave, as well as infestation of pest and diseases. These adverse impacts on oil palm production highlight the significance of deploying effective adaptation strategies. The study aims to examine the impact of climate change on oil palm production and identify the farmers’ adaptation strategies to the impacts of climate change in Malaysia. This study was conducted a comprehensive review of the articles published from 2000 to 2021 in the contexts of climate change and oil palm production in Malaysia. The review shows that climate change has a range of impacts on the oil palm production in Malaysia. As a result, several adaptation options were identified, such as breeding of hybrid varieties that are tolerant and resistant to heat; sustainable management of soil; pit and tranches to enhance water management in plantation areas; minimal use of fertilisers, herbicides, and pesticides; zero burning; and minimum tillage. The reviewed studies recommended the following to mitigate the adverse impacts of climate change: sustainable national policy on climate change, conservation of the existing carbon stock, effective management of tropical rainforest biodiversity, afforestation for carbon sequestration, and reduction in greenhouse gas (GHG) emission.

## Introduction

Weather denotes the present condition of the atmosphere, whereas climate reflects the average weather condition of a geographical location for over 35 years (Kevin et al. 2000). Climate change is a change in the state of the climate identified (e.g. using statistical tests) through changes in the mean and/or the variability of its properties that persists for an extended period, typically decades or longer (Intergovernmental Panel on Climate Change [IPCC] 2007). As a global challenge to the economy especially sectors that highly rely on climate (e.g., agriculture) (Vaghefi et al. 2016), climate change is strongly linked with agriculture and food production (Zimmermann et al. 2018).

The rapid expansion and conversion of tropical rainforests to oil palm plantation have manifested in low-cost oil palm production from land preparation to harvesting, thus making the crop a profitable commodity with high turnover and economically viable (Paterson and Lima 2018). The palm oil business attracts investments worth over USD 50 billion annually (Murphy 2014; Paterson and Lima 2018). Crude palm oil (CPO) is processed and used for various purposes, ranging from production of chemicals, detergents, and biodiesels (Kamil and Omar 2017; Rival 2017; Paterson and Lima 2018; Zahan and Kano 2018). However, there has been no consensus from the scientific debates pertaining to the impacts of oil palm expansion (Tang and Al Qahtani 2020). According to some quarters, oil palm expansion alleviates the impacts of climate change by providing biofuel as an alternative energy, apart from contributing to Gross Domestic Production (GDP) and local livelihood (Basiron 2007). On the other hand, some are concerned about environmental degradation and destruction of natural forest, claiming no any other human activity that destroys the natural system of the earth than agriculture (Foley et al. 2005; Barcelos et al. 2015; Obidzinski et al. 2012; Devendra 2012).

The climate in Malaysia is characterised by the changing pattern of rainfall in duration and intensity (Tangang et al. 2012; Tangang et al. 2017; Tangang et al. 2018; Tang 2019). Higher rainfall variability was recorded since the last two decades (Tang 2019). The increasing number of hot years clearly signifies a change in the climate (Tangang et al. 2012; Shahid et al. 2017). Glacier, ice sheets, and frozen grounds appear to be melting at an alarming rate, along with the thermal expansion of the ocean (IPCC 2007; Pachauri and Reisinger 2007; Abdul Rahman 2018). These have led to the combined effects of rising ocean level, waves, and tides (Tangang et al.^2012^; Cazenave and Cozannet 2014). Malaysia was worst hit by flood during 1997–1998 and 2006–2007, which was devastating as these adversities culminated many lives and properties (Al-Amin et al. 2011).

Therefore, addressing the impacts of climate change on oil palm production and productivity demands the development of sound adaptation strategies (Tang and Al Qahtani 2020; Ahmed et al. 2021). These policies should have focus and direction based on the changes, as well as anticipations brought about by new policies, market preferences, and unexpected changes in weather conditions that occur regularly (Suresh 2013). The adaptation process should, however, either be reactive in response to the current climatic challenges or be anticipatory through standby or deployed before the unexpected occurs (Füssel 2007; Holman et al. 2019; Rahman and Hickey 2019). These may be achieved through debated policies decisions and engagement of stakeholders (Nguyen Long et al. 2019). The adaptation process should weigh in current and future technological changes, land tenure systems, food scarcity, and sustainable ecosystem management (Thornton et al. 2018; Sarkar et al. 2020). Both the nature and the degree of adaptation relies on the knowledge of the present and future climate (Gornall et al. 2010; Arbuckle et al. 2015). The correlation of climates in the next decades, and the adaptive capacity of farmers are integral for adaptation to climate change, particularly in the Southeast Asia region where fluctuation and variability are highly expected (Ernawati Hamdan et al. 2013; Tang 2019). Some successful adaptation strategies implemented in the oil palm sector are listed as follows: silt pit tranches for water conservation, soil and land management, carbon sequestration, zero burning, minimum chemical inputs to mitigate GHGs emission, enhanced water management in the plantation, reduction in the number of oil palm fronds (OPFs), intercropping cropping, diversification, use of improved variety and sustainable pest, as well as effective management of diseases (Murtilaksono et al. 2011; Bohluli et al. 2014; Nabara and Man 2018; Sarkar et al. 2020; Ahmed et al. 2021). These may be influenced and changed if the current climate trends continue, mostly depending on the climate change scenarios, agroecological practices, and geographical location (Suresh 2013; Darras et al. 2019; Sarkar et al. 2020).

The objective of this study is to examine the impact of climate change on oil palm with reference to temperature, rainfall, and extreme weather events (El Niño, La Niña, drought and flooding), and identify the farmers’ adaptation strategies to the impacts of climate change in Malaysia. Studies on oil palm and climate change in Malaysia have mostly assessed socioeconomic impacts of climate change (Al-Amin et al. 2011; Liew et al. 2014; Shanmuganathan et al. 2014; Sani et al. 2014; Zimmermann et al. 2018; Tang 2019; Sarkar et al. 2020), biodiversity and environmental sustainability (Weng 2005; Fitzherbert et al. 2008; Siwar et al. 2009; Zulkifli et al. 2010, 2017; Dayang Norwana et al. 2011; Fold and Whitfield 2012; Lane 2012; Murphy 2014; Savilaakso et al. 2014; Barcelos et al. 2015; Pirker et al. 2016; Varsha et al. 2016; Tang and Al Qahtani 2020), oil palm diseases (Paterson et al. 2013; Paterson and Lima 2018; Paterson 2019), effects of edaphic factors on oil palm production (Weng 2005; Khalid and Tarmizi 2008; Bakar et al. 2011; Mohsen et al. 2014; Behera et al. 2016; Afandi et al. 2017), and oil palm adaptation strategies to climate change (Alam et al. 2013; Hamdan et al. 2013; Bessou et al. 2017; Nabara and Man 2018; Man et al. 2019; Hosen et al. 2020). Despite being the largest agricultural contributor to Malaysia’s economy, oil palm recorded fewer studies than paddy rice (Tang 2019).

This study, hence, accentuates the current trends and projections of climate change on oil palm cultivation in Malaysia and provides a strategic overview of its impacts, adaptation and mitigation. It is important to understand the past climate in order to adjust the present and plan for the future. Therefore, this study provides useful information for policymakers in the oil palm sector in Malaysia and enables formulation of sustainable policies to determine the best adaptation and mitigation measures. Meanwhile, the study contributes to the understanding of the impacts of climate change and adaptation on oil palm production and filled the existing gaps identified in the literature.

The study involved systematic literature review. Articles related to this study were retrieved from reputable databases, such as Scopus, Elsevier, ProQuest, ResearchGate, and Google Scholar. The documents were accumulated from search engines using relevant search terms, including “climate change”, “oil palm”, “climate change and oil palm”, “oil palm and extreme events”, and “oil palm adaptation”. The abstract of the retrieved documents was extensively reviewed for categorisation into a range of themes and associations. At this stage, duplicate documents were discarded, thus leaving only the relevant original documents for further review. Articles written other than the English language and published before year 2000 were excluded. Articles reviewed in this study were selected as indicated in their title or abstract pertaining to adapting and mitigating both direct and indirect impacts of climate change. Besides, full-text review and assessment of documents that report adaptation and adaptive capacity were included as well. On top of that, articles that reported challenges faced due to changing climate, perceived impacts of extreme events on oil palm, temperature, and rainfall variability, resilience to climate change, as well as vulnerability and projection of future climate, were incorporated.

### Oil palm agronomy and brief history

A member of the *Palmea* family (*Elaeis guineensis*), the oil palm contains single seed or monocotyledon and fibrous roots system spreading downward in search of nutrients, and for single trunk erection (Corley and Tinker 2015; Ekenta et al. 2017). The crown of oil palm consists of 25–40 matured fronds (Ekenta et al. 2017). The native of West and Central Africa was imported to Malaysia by the British colonial in 1800s, wherein the first commercial oil palm plantation was established in Selangor in the year 1917 (Zulkifli et al. 2010; Zaki and Rahim 2019). A palm oil tree grows to about 10 m tall and has a life span of 25–30 years prior to replanting (Corley and Tinker 2003). Oil palm harvest, known as fresh fruit bunch (FFB), is composed of mainly oil (25%), kernel/seed (5%), mesocarp fibre (13%), shell (7%), and empty fruit bunch (EFB) (23%) (Corley and Tinker 2003; Zulkifli et al. 2010). Oil palm trees are planted in polyethene bags (15 × 23 cm) with protective cover for 3–4 months after germination (Corley and Tinker 2003; Zulkifli et al. 2010).

### Oil palm plantation

Large-scale oil palm plantation demands mechanical means of land clearing and preparation due to massive area coverage (Mutsaers 2019). The use of heavy machines destroys not only the soil structure and texture, but also the cation exchange capacity, which exposes soil to the direct impacts of sunlight (Corley and Tinker 2003). Land clearing via bush burning releases CO_2_ that destroys macro and microorganisms in the soil, which facilitate in decaying and decomposition of organic matter, aeration, and pollination (Dislich et al. 2017). These combined effects of mechanical land clearing and fire lead to hardening and compaction of soil that destroy essential nutrient elements, render them unavailable for plant use, and degrade ground vegetation—ultimately changing the characteristics of the entire ecosystem (Dislich et al. 2017). Several laws have been enacted against land clearing using fire, example, National Forestry Act 1984 (amended 1993) (Abdullah 2002; Diemont et al. 2002). Despite that, bush burning remains a common land clearing practice among oil palm producers in Malaysia (Seng 2017). Oil palm demands less application of fertiliser than paddy rice, cotton and wheat (Behera et al. 2016; Ahmed et al. 2021). The heavy feeder nature of the oil palm requires optimum and balance application of fertiliser to supplement lost soil nutrients for maximum yield (Behera et al. 2016). Oil palm is the most economically efficient oil crop at the global level, simple to establish, low production cost, and high yield turnover (Basiron and Weng 2004; Dislich et al. 2017; Paterson and Lima 2018; Kushairi et al., 2018).

### Oil palm production

Oil palm production in Malaysia involves large-scale and smallholder plantations (Ahmed et al. 2021). Large-scale plantation is defined by its area extent of 3000–20000 ha (Sheil et al. 2009). In total, 423 functional palm oil processing factories are established in Malaysia (Chiew and Shimada 2013). The expansion of oil palm plantation and setting up of large processing companies maximise profit at low cost and minimal labour (Herdiansyah et al. 2020). These companies are more concerned about high yielding and early maturing oil palm variety seeds (Durand-Gasselin and Cochard 2005). Meanwhile, smallholders do not have adequate capital to access high yielding variety seeds and might not be able to differentiate local seeds from the improved variety (Zen et al. 2006).

### Oil palm–planted area

**Fig. 1:**
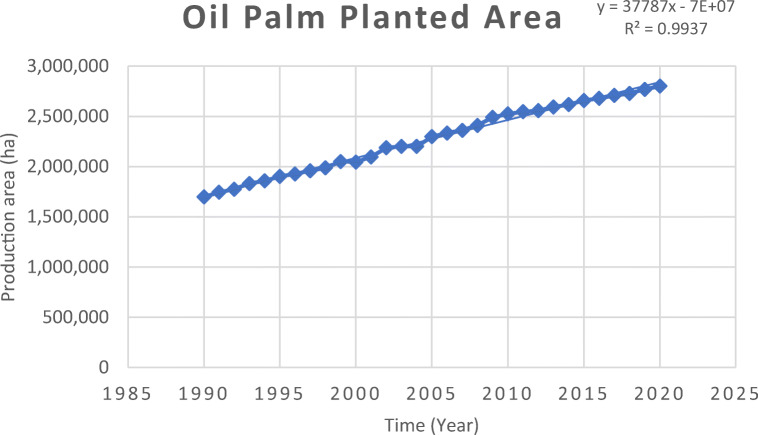
Trend of oil palm planted area in Malaysia 1990–2020 MPOB (2020)

**Fig. 2:**
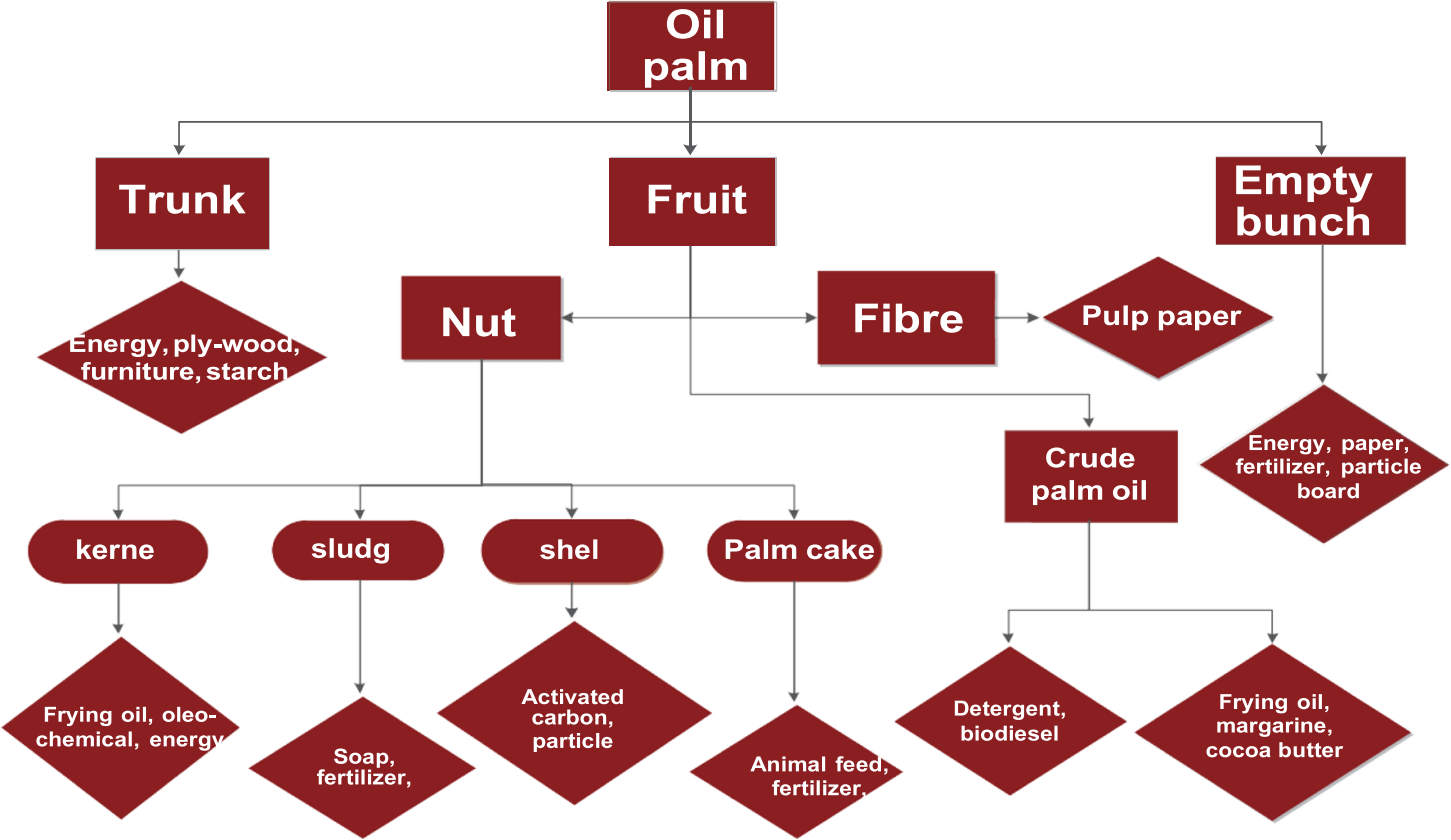
Trend of fresh fruit bunches (FFB) yield in Malaysia 1990–2020 MPOB (2020)

**Fig. 3:**
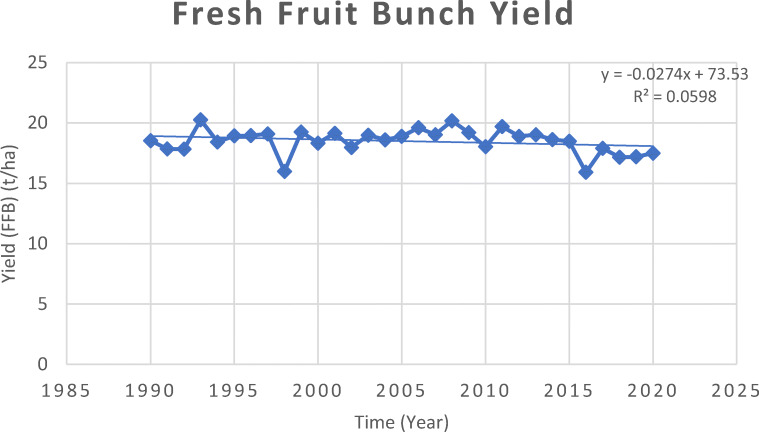
Oil palm product value chain. Source: Ministry of Food and Agriculture (2010)

**Fig. 4:**
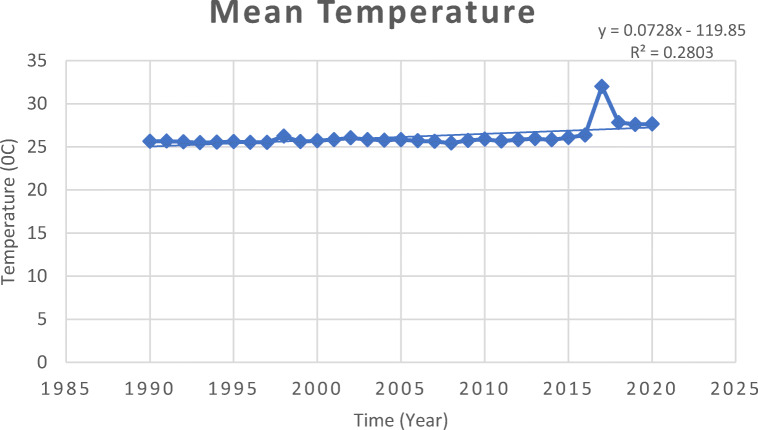
Trend of mean temperature in Malaysia 1990–2020. Source: World Bank Climate Knowledge Portal, 2021

**Fig. 5:**
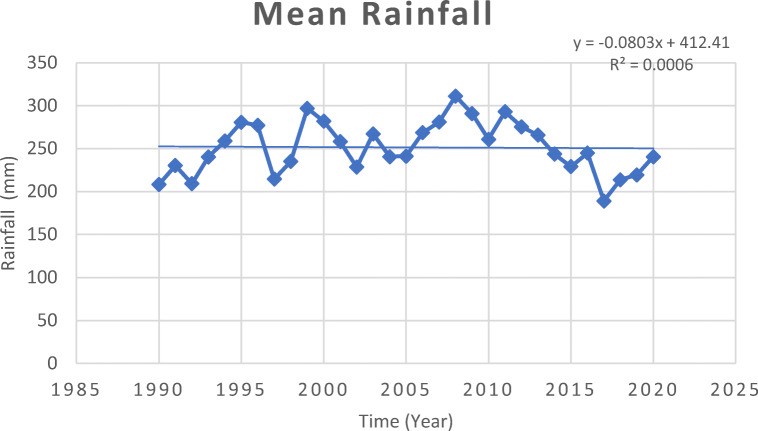
Trend of mean rainfall in Malaysia 1990–2020. Source: World Bank Climate Knowledge Portal, 2021

The cultivation of oil palm is spread across lowland of the tropics (18.1 million hectares across 43 countries) (Dislich et al. 2017). Indonesia is the largest oil palm producer accounting for 7.1 million ha, while Malaysia at 5.9 million ha (Dislich et al. 2017; MPOB 2020). Notably, both these countries contribute to 85% of worldwide CPO production (Wahid et al. 2006; Dislich et al. 2017). As a result of the agricultural transformation policy in Malaysia, more land was converted from rubber to oil palm during the 1960s and 1970s, resulting in a significant increase in the oil palm planted area (McCarthy and Cramb 2009; Nambiappan et al. 2018; Shevade and Loboda 2019). In recent years, most of the expansion took place in Sabah and Sarawak due to declining availability of suitable land in Peninsular Malaysia (Nambiappan et al. 2018; Shevade and Loboda 2019). In 2016, about 47% of the planted area is in Peninsular Malaysia, 27% in Sabah and 26% in Sarawak (Nambiappan et al. 2018). Overall, in Malaysia the trend of oil palm planted area is on increase (MPOB 2020) (Figs 1-5).

### Fresh fruit bunch production

In 1990, the average FFB yield was 18.53 tonnes per hectare, ranging from 17.83 tonnes to as high as 20.26 tonnes per hectare (MPOB 2020). The average FFB yields recorded in 2014 was 18.65 tonnes whereas in 2019, average yields recorded was at 17.19 tonnes per hectare in Malaysia (MPOB 2020). Malaysia has seen a significant variation in yearly yields, with marked interannual rises and drops over the last 30 years (1990-2020) (MPOB 2020; MPOC 2020). The high to optimum FFB yields are obtained under ideal climatic conditions, with at least 2000–3000 mm of rainfall distributed evenly throughout the year and there should be more than 200 rainy/days/year (Chantaraniyom 2007; Carr 2011; Unjan et al. 2017; Oettli et al. 2018). The relative humidity should be between 75 and 80%, average temperature of 25°C, and more than 5.5 h of sunlight per day (Chantaraniyom 2007; Carr 2011; Eksomtramage 2011; Unjan et al. 2017; Oettli et al. 2018).

### Oil palm value chain

Some of the by-products (e.g. mill effluent and EFB) are returned to the soil for mulching and enhancing soil fertility (Ahmed et al. 2021). Shell is spread in rows within the plantation and also used in building and construction (The Star 2010; Ahmed et al. 2021). Fibre and palm kernel shell provide energy to the palm oil processing mills (Yusoff 2006). The trunk is modified through industrial processes to produce furniture (Simorangkir 2007; Sheil et al. 2009). Other products derived from oil palm are paper, fibre board, cosmetics, plywood, domestic cooking oil, and animal feed. (Wahid et al. 2006; Paterson et al. 2017; Paterson 2020a). Industrial recycling of palm oil by-products reduces wastage and environmental degradation, creates employment, improves the purchasing power of the producers, and significantly contributes to the GDP of Malaysia (Shamsuddin et al. 2021).

### Palm oil exports

Palm oil and kernel are the two major commodities extracted from oil palm for export (MPOB 2011). Approximately half of the world demand for palm oil products supplied by Malaysian exports (Sumathi et al. 2008). In 2009, a rise of 2.8% in the exports of oil palm products including palm oil, palm kernel oil, palm kernel cake, oleo-chemicals, biodiesel, and finished products was recorded (MPOB 2011). Malaysian exports of palm oil are more than 17 million metric tonnes annually (MPOC 2021). Data from 2020 shows that Malaysia exported 12.95 million metric tonnes of processed palm oil (PPO) and 4.42 million metric tonnes of CPO for that year (MPOC 2021). Exports of CPO is projected to rise in 2021 to about 5.5 million metric tonnes, which would be an increase of 24.4% year to year from 2020; this is due to CPO from Malaysia becoming more competitive in prices as the government of Indonesia has revised its export duty policy (MPOC 2021). The CPO production in Malaysia is also projected to rise to 19.6 million metric tonnes in 2021 from 19.14 million metric tonnes in 2020, which is a moderate increase (MPOC 2021). In 2020, the major oil palm export destinations from Malaysia were China (2,730.66 metric tonnes), India (2,726.66 metric tonnes), the Netherlands (1,072.95 metric tonnes), Pakistan (1,003.72 metric tonnes), the Philippines (693.03 metric tonnes), Turkey (615.87 metric tonnes), the USA (540.35 metric tonnes), Kenya (520.76 metric tonnes), South Korea (453.76 metric tonnes), and Italy (439.05 metric tonnes) (Statista 2021) (Tables 1-7).

**Table 1:** Exports of all palm products in Malaysia

Product	Unit	Jan–Dec 2020	Jan–Dec 2019	Change (Mt)	Change (%)
CPO	MT	4,423,694	3,827,915	595,779	15.56
	RM Million	12,101.53	8,019.42	4082.11	50.90
PPO	MT	12,945,171	14,643,150	(1,697,979)	(11.60)
	RM Million	36,792.80	33,628.59	3164.21	9.41
Palm oil	MT	17,368,865	18,471,065	(1,102,200)	(5.97)
	RM Million	48,894.33	41,648.01	7246.32	17.40
CPKO	MT	416,836	334,179	82,657	24.73
	RM Million	1390.84	855.59	535.25	62.56
PPKO	MT	802,856	752,075	50,781	6.75
	RM Million	3051.23	2710.61	340.62	12.57
Palm kernel oil	MT	1,219,693	1,086,254	133,439	12.28
	RM Million	4442.06	3566.20	875.86	24.56
Palm kernel cake	MT	2,568,704	2,492,738	75,966	3.05
	RM Million	1362.05	1016.91	345.14	33.94
Oleo chemicals	MT	3,058,031	3,280,127	(222,096)	(6.77)
	RM Million	12,467.56	12,299.69	167.87	1.36
Finished products	MT	561,279	593,714	(32,435)	(5.46)
	RM Million	2610.75	2558.38	52.37	2.05
Biodiesel	MT	378,582	609,777	(231,195)	(37.91)
	RM Million	1354.54	1603.77	(249.23)	(15.54)
Others	MT	1,500,240	1,345,502	154,738	11.50
	RM Million	1634.79	1038.23	596.56	57.46
Total	MT	26,655,394	27,879,177	(1,223,783)	(4.39)
	RM Million	72,766.09	63,731.19	9034.90	

Source: Malaysian Palm Oil Council [MPOC] 2020. Note: Metric tonne (MT), Malaysian ringgit (RM), crude palm oil (CPO), processed palm oil (PPO), crude palm kernel oil (*CPKO*), processed palm kernel oil (PPKO)

**Table 2:** Sea level rise projection in Malaysia

	Malaysia	Global
Mareograph data	2.2-5.3 1993–2015	3.2 (2.8–3.6)
Altimeter data	2.8-4.4 1993–2015	
		IPCC (AR5, 2013)
(RCP 8.5) 2100 sea level rise projections for Malaysia		
	Sea level rise	Note
Peninsular Malaysia	0.6–0.71 m (10.5–10.9 mm/year)	Maximum surge in sea level rise at the east coast region in Peninsular Malaysia (Kelantan, Johor, Terengganu, and Pahang)
Sabah and Sarawak	0.71–0.74 m (10.9–11.1 mm/year)	Maximum surge in sea level rise in Kudat, Sabah

Source: Bilik (2019)

**Table 3:** Crop type and yield in respond to changing temperature in Southeast Asia

Region	Temperature	
Southeast Asia	+2°C C3 crops---------C4 crops −23.71 −-23.71	+ 4°C C3 crops---------------C4 crops −43.60 −43.60

Source: Calzadilla et al. (2013) cited in Sarkar et al. (2020). Note: cereals, vegetables, and oil seeds are C3 crops; while sugar cane and cereal grains are C4 crops

**Table 4:** Biotic and abiotic factors that affect oil palm yield

Biotic and abiotic factors	Anticipated changes/outcome for the 21^st^ century	Expected outcome on oil palm yield
Average annual rainfall	Influenced by local geographical differences. Increase in some locations, while decrease in others.	Heavy rainfall without prolong flooding increased yield turnover, while low rainfall depicted low yield harvest (FFB)
Uncertainty and rainfall variability	Low rainfall accompanied with droughts and frequent flooding	Yield decline drastically
Temperature variability	Increase	High evapotranspiration, salinity and dry soil
Carbon dioxide concentration (CO_2_)	Increase	High yield
Sea levels	Increase	Damages at coastal plantation and incurred losses
Pests and diseases	Increase	Damages oil palm and affects yield
Pollinators	Decrease	Reduction in yield

Source: Fleiss et al. (2017)

**Table 5:** The losses in oil palm plantations for each return period

Flood events (years ARI)	Total area (ha) affected	Total FFB (tonnes)	Total price loss (million RM)
10	1759.97	2446.36	1.55
50	3642.32	5062.82	3.21
100	4249.62	5906.97	3.75

Muhadi et al. (2017)

**Table 6:** Historical impact of El Niño on palm oil yield and production in Malaysia

Types	Period	Intensity	Change in FFB yield	Change in CPO yield	CPO output growth
El Niño	May 82–June 83	Strong	−10.5%	−10.4%	−14.1%
El Niño	Aug 86–Feb 88	Moderate	−22.8%	−23.1%	−0.2%
El Niño	May 91–June 92	Moderate	−0.1%	−1.4%	3.8%
El Niño	Sep 94–May 95	Moderate	2.8%	2.0%	8.2%
El Niño	May 97–April 98	Strong	−16.3%	−16.8%	−8.3%
El Niño	May 02-Feb 03	Moderate	5.7%	4.5%	12.1%
El Niño	Jul 04–Jan 05	Weak	1.5%	1.9%	7.1%
El Niño	Sep 06–Jan 07	Weak	−2.9%	−2.5%	−0.4%
El Niño	Jul-09-Apr 10	Moderate	−6.1%	−6.1%	−3.3%
El Niño	Mar 2015–May 2016	Strong	−13.2%	−3.21%	8.0%

Ivy-Ng (2015) and Yong (2017)

**Table 7:** Summary of the major existing studies regarding the impacts of climate change on oil palm plantation in Malaysia

Author and year	Title of the article	Significant findings
Sarkar et al. (2020)	Impacts of climate change on oil palm production in Malaysia.	A negative relationship was noted between annual temperature and oil palm production. If temperature rose by 1–4 °C, the oil palm yield declined to 10–40%.
Oettli et al. (2018)	Climate based predictability of oil palm tree yield in Malaysia.	The occurrence of El Niño in the Pacific Ocean reduced the amount of rainfall received over Malaysia and increased the air temperature. These caused water stress to oil palm trees and decreased FFB yield.
Nabara and Man (2018)	The role of extension activity-based adaptation strategies toward climate impact among oil palm smallholders in Malaysia: A systematic review.	The use of planting materials tolerant to extreme rainfall, soil, and water conservation, as well as extension programmes, appears to be an effective adaptation measure to climate change.
Nda et al. (2018)	Effects of hydrological parameters on palm oil FFB yield.	Variability of temperature and rainfall from one month to the other had a significant impact on oil palm yield.
Chizari et al. (2017)	Economic climate model of the oil palm production in Malaysia.	Rising temperature and decreasing precipitation reduced oil palm yield.
Wan Noranida et al. (2015)	Effect of climate change on oil palm production in Malaysia.	Increment in temperature adversely affected oil palm yield.
Paterson et al. (2015)	Future climate effects on suitability for growing of oil palm in Malaysia and Indonesia.	A significant decrease was noted in climatic suitability for oil palm production, particularly a gradual drop in climatic suitability by 2030 and a rapid decrease by 2100. This implies that oil palm production will be affected severely.
Paterson et al. (2013)	How will climate change affect oil palm fungal diseases.	The climate favours the emergence of new oil palm diseases. Changing climate modified the pathogenic distribution range, temporal activity, and community structure.
Paterson and Lima (2018)	Climate change affecting oil palm agronomy, and oil palm cultivation increasing climate change, require amelioration.	A significant reduction in climatic suitability for oil palm production due to climate change and variability.
Zainal et al. (2012)	Economic impacts of climate change on the Malaysian palm oil production.	Climatic change displayed nonlinear impacts on net revenue, apart from predicting a drop in revenue from the oil palm sector.

## Climate change and extreme weather events

### Temperature

The last century witnessed the rise in the global average temperature by 0.3–0.6°C (Tangang et al. 2007). In fact, the warming trends have continued rising by 0.15°C per decade since 1970 (Feidas et al. 2004; Quadir et al. 2004; Tangang et al. 2007). The annual variation of mean temperature from 1980 to 2002 was 0.5–2.0°C (Ariffin et al. 2003). The lowest recorded value was obtained in December–January during the northeast monsoon (Information Bulletin 2017), whereas the maximum temperature was recorded in Kota Bharu with the mean temperature of 28°C (Tangang et al. 2007). Records obtained from 23 meteorological stations in Malaysia signified an increase in daily mean average temperature above 28°C and a greater number of hotter days (Tangang et al. 2017; Tang 2019). Most of the meteorological stations revealed positive trends of extreme temperature beyond mean annual average of 26–28°C (Hanim et al. 2015; Ministry of Energy, Science, Technology, Environment and Climate Change [MESTECC] 2018).

Temperature projections for Sabah and Sarawak using Regional Hydroclimate Model for Sabah and Sarawak (RegHCM-SS) predicted rise in average annual temperature by 1.5°C and 3.7°C for 2040–2050 and 2090–2100, respectively (Shaaban 2013). In particular, Sarawak was projected to record mean annual temperature of 1.23°C and 3.10°C for 2040–2050 and 2090–2100, respectively (Shaaban 2013). Based on the General Circulation Model from 1961 to 1999, the rising temperatures could range at 1.0–3.5°C and 1.1–3.6°C until year 2095 for Malaysia Borneo and Peninsular Malaysia, respectively (Malaysian Meteorological Department 2009). Warmer days are predicted to increase by 28–75% and warmer nights by 46–95% by 2100 (Kwan et al. 2011). Although Peninsular Malaysia seemed to likely less suffer from warming (Kwan et al. 2011; Abdul Rahman 2018; Tang 2019). Similarly, temperature change patterns indicate a nationwide warming over Malaysia (Tangang et al. 2012; Abdul Rahman 2018). Malaysia is expected to become hotter by 2050, with temperatures rising by up to 1.5°C (Abdul Rahman 2018). The projected temperature rises by the end of the twenty-first century in Malaysia ranges from 2.5–3.9°C, 2.7–4.2°C, and 1.7-3.1°C, respectively (Loh et al. 2016).

### Sea level rise

Global warming and melting ice have led to rising ocean sea level, apart from exacerbating the impact of coastal inundation (Church et al. 2013). Nonetheless, year 2015 witnessed a decline in the average sea level rise in Lahad (Sabah), despite the general increasing trend for mean sea level in Malaysia at 3.67± 0.15 mm per year from 1984 to 2013 from the tidal data (Kamaruddin et al. 2016; Bilik 2019). The projections for Peninsular Malaysia and Malaysia Borneo sea levels in year 2100 indicated rise at 0.517 m and 1.064 m, respectively (Ercan et al. 2013; Kamaruddin et al. 2016). About 1,000,000 ha and 80,000 ha of oil palm and rubber land would be lost over 1 m rise in sea level (Chong 2000; Al-Amin et al. 2011).

### Rainfall

The trends of rainfall appeared to vary between 1990 and 2020 particularly in Peninsular Malaysia and Sabah (MMD 2009). The historical meteorological data highlighted vivid variation in rainfall pattern over years (MMD 2009; Sammathuria and Ling 2009; Zainal et al. 2012; Loh et al. 2016; Tang 2019). The increase in rainfall intensity and duration was attributed to the northeast monsoon featuring strong northeasterly wind originating from the Tropical Western Pacific towards the South China Sea between November and February, accompanied with wet moisture in the entire region (Chang et al. 2005; Ayat et al. 2013). Kwan et al. (2011) projected changes in increasing frequencies of overwhelming rainfall especially during northeast monsoon and towards the last quarter of every year. Rainfall is anticipated to increase by ~20 to 40% during summer across the Southeast Asia region, as portrayed by the Intergovernmental Panel on Climate Change (IPCC) Special Report on Emission Scenarios (SRES A2, A1B and B2) (Loh et al. 2016). Syafrina et al. (2017) projected increase in hourly duration and intensity of rainfall for the period 2081–2100 with spatio-temporal variation in the distribution within the Peninsular Malaysia based on the Representative Concentration Pathway (RCP 6.0) scenario.

### Drought

Water scarcity will be a big issue for Peninsular Malaysia in the twenty-first century and is likely to be a major environmental problem in the whole country (Fung et al. 2020). In theory, major shortfalls in rain will result in a drought situation; water sufficiency will be severely affected, and agricultural and human activities will face the challenges of water scarcity (Wilhite et al. 2014; Fung et al. 2020). A lengthy drought skews the supply and demand balance of water, which results in a significant increase in oil palm vulnerability to adverse effects (Payus et al. 2020). Extreme drought results in dry weather that is abnormal, which seriously unbalances water cycles and changes the processes of precipitation and evaporation, atmospheric water vapour circulation, and soil moisture availability (Zhang et al. 2014; Hasan et al. 2021). Malaysia previously experienced its most severe droughts during the hydrological years of 1996/1998, 1997/1998, 2002/2003, and 2016/2018 (Hasan et al. 2021).

### Flood

Flood disasters have become more frequent and severe as a result of anomalous changes in air temperature and heavy rainfall (Hazran et al. 2017). Floods have adverse effects on oil palm cultivation, and the effects can last for long time (Sani et al. 2014). In the coming decades, climate change is expected to make the situation even more difficult. Floods and flash floods are common in Malaysia during periods of persistent rainfall which accounts for significant losses (Safiah Yusmah et al. 2020). Flooding is common during the northeast monsoon season often experienced in the east coast states of Peninsular Malaysia such as Kelantan, Terengganu, Pahang and Western Sarawak (Buslima et al. 2018). The estimated area prone to flood disaster is approximately 29,800 km^2^ of the total land area in Malaysia, and it affects nearly 4.82 million people in the country (Department of Irrigation and Drainage 2009; Sani et al. 2014; Mohd Taib et al. 2016). Flood risk, exposure, and damage potentials are increasing, contributing to an increase in poverty and vulnerability. The annual occurrence of the flood hazard has compelled oil palm growers to plan ahead of time in order to minimise its impacts (Safiah Yusmah et al. 2020). Large floods with devastating impacts that occurred in Malaysia include 1886, 1926, 1931, 1947, 1954, 1957, 1965, 1967, 1970/1971, 1988, 1993, 1996, 2000, 2006/2007, 2008, 2009, and 2010 (Buslima et al. 2018).

### El Niño

The El Niño Southern Oscillation happens from a series of interaction between atmosphere and oceans, particularly in the Tropical Pacific (Oettli et al. 2018). This may affect different areas globally at different time of the year or over years (Abdul Rahman 2009). The tele-connected impact results from regional changes in air and sea surface interaction, as well as alteration of Walker circulation (Tangang et al. 2012; Sum 2018; Tangang et al. 2018; Koplitz et al. 2018). El Niño is characterised by abnormally warm ocean temperatures in the Equatorial Pacific (Kamil and Omar 2017). The El Niño had caused devastating environmental and socioeconomic impacts to Malaysia (Tangang et al. 2012; Oettli et al. 2018; Sum 2018). The phenomenon had a greater impact in Sabah and Sarawak than in Peninsular Malaysia (Kamil and Omar 2017). The historical El Niño events in Malaysia include that of 1951/52, 1953/1954, 1957/58, 1965/66, 1969/70, 1972/73, 1977/78, 1982/83, 1986/87, 1991/92, 1994/95, 1997/98, 2000/01, 2003/05, 2007/08, 2014/15, and 2015/16 (Ariffin et al. 2003; Tangang et al. 2017; Filho et al. 2018). The strongest El Niño on record were that of 1982/83, 1997/98, and 2015/2016 (Tangang et al. 2017; Filho et al. 2018). The recent El Niño of 2015/

16 was slightly stronger, in terms of sea surface temperatures, than that of 1982/83 El Niño, but weaker than 1997/98 episode (Kamil and Omar, 2016; Sum 2018). A study based on the Princeton Ocean Model revealed that extensive years of climate analysis over the Southeast Asia region indicated extreme weather events around the Sunda Shelf, which may continue along with increasing intensity and momentum towards the Gulf coast area of Thailand and Malaysia (Wah et al. 2012).

### La Niña

On the contrary, La Niña, the “girl child” signified extreme cooling at the Eastern, the Central Pacific, and the entire Equatorial Zone (Ayat et al. 2013). While the climatic phenomenon usually peaks in intensity between October and January, causing changes to climatic patterns and their related impacts on oil palm cultivation (Oettli et al. 2018). During La Niña events, the Central-to-Eastern Equatorial Pacific is colder than usual, inhibiting the formation of rain-producing clouds while increasing atmospheric convection and rainfall in the Western Equatorial Pacific (Cai et al. 2015). As a result of the 1998/1999 La Niña event, about 25,000 to 50,000 people lost their lives in Venezuela (Takahashi et al. 2001; Cai et al. 2015). Storms in China killed thousands and displaced over 200 million people (Jonkman 2005; Cai et al. 2015). Bangladesh experienced one of the most devastating overflow events in recent times, with more than half of the total land area of the country inundated, resulting in severe food shortages and the spread of waterborne epidemic diseases, killing several thousand people and affecting over 30 million more (Del Ninno and Dorosh 2001; Kunii et al. 2002; Mirza et al. 2002; Cai et al. 2015). The consequences of La Niña on oil palm production can be both positive and negative (Ayat et al. 2013; Kamil and Omar 2017). La Niña brought about wetter condition in the Southeast Asia and its frequency was lower than El Niño (Ariffin et al. 2003). The La Niña years in Malaysia include the 1950/51, 1955/56, 1970/71, 1973/74, 1975/76, 1988/89, 1998/99, and 2000/01 (Arrifin 2003; Tangang et al. 2017; Filho et al. 2018).

### Climate change impacts on oil palm production

Climate change has profound impacts on oil palm production and this has been extensively discussed in the literature (Basiron and Weng 2004; Abul Quasem et al. 2011; Shanmuganathan and Narayanan 2012; Ab Rahman et al. 2013; Paterson et al. 2017; Paterson and Lima 2018; Nabara and Man 2018; Oettli et al. 2018; Paterson 2019, 2020a, b; Sarkar et al. 2020). Climate change has more negative impacts on oil palm production than positive impacts (Sarkar et al. 2020). These impacts include rising temperature, water stress, infestation of pest and diseases, yield reduction, and decline in revenue (Sarkar et al. 2020).

### Impact of temperature on oil palm production

The increment in average global temperature by 0.85 °C from 1880 to 2012 had affected the agricultural settings across many nations (IPCC 2014; Allen et al. 2018). The scientific literature depicts that temperature variability can be unfavourable for production of oil palm and other crops (Sarkar et al. 2020; Ahmed et al. 2021). Different crops respond differently to varying degree of temperature, thus affecting yield turnover (Calzadilla et al. 2013; Othman and Jafari 2014; Sarkar et al. 2020).

Increase in temperature by 2 °C, could result in 30% shortfall in oil palm yield (Ministry of Natural Resources and Environment 2010; Paterson and Lima 2018). Rise in temperature by 1–4 °C could decline 10–40% of oil palm yield (Sarkar et al. 2020). Zainal et al. (2012) reported that CPO production had dropped slightly from 17.00 metric tonnes to 16.17 metric tonnes in 2009 due to the effects of temperature variability. Accordingly, CPO production declined by 6.1% (9.5 MT) per hectare in Peninsular Malaysia and 2.5% (5.3 MT) per hectare, in Sabah (Malaysian Palm Oil Board 2010; Zainal et al. 2012). The combined effects of temperature variability had affected the production of FFB by 6.1% and a slight decline of 0.2% in Oil Extraction Rate for the year 2009 (MPOB 2010). Accordingly, Peninsular Malaysia, Sabah, and Sarawak recorded a decline in FFB at 7.5%, 4.7%, and 2.6%, respectively (MPOB 2010). Temperature variability has a significant nonlinear impact on oil palm net revenue in Malaysia. The total marginal increase in temperature resulted in losses of RM 40.55 million, RM 48.69 million, and RM 37.61 million ha^−1^ for Peninsular, Sabah, and Sarawak, respectively (Zainal et al. 2012). Similarly, by the years 2029, 2059, and 2099; oil palm revenue would decline by RM341.29 (RM ha^−1^) for Peninsular Malaysia, RM 127.43 (RM ha^−1^) for Sabah, and RM 51.80 (RM ha^−1^) for Sarawak (Zainal et al. 2012; Paterson and Lima 2018).

Heatwaves and temperature are on increase in Malaysia (Filho et al. 2018). Increment in temperature accelerates the rate of soil water evaporation, thus making the soil drier and worsening the impacts of water scarcity on oil palm. The temperature projection for Southeast Asia in 2100 would be extreme for oil palm (Dumbrell and Hill 2005; Paterson et al. 2015; Fleiss et al. 2017). Wen and Sidik (2011) claimed that any minimal rise in temperature may increase the yield harvest, as noted in some coastal areas along Sabah. Increase in temperature favours the ecology of pests and diseases, as well as changes in their fecundity (physiological ability to reproduce) (Fleiss et al. 2017). The pollination activity of *Weevil* species may be reduced due to temperature rise (Jackson et al. 2010). Other pollinators, including *E. kamerunicus* (a native of Africa brought to Southeast Asia) could be at danger or risk of diseases that might severely reduce the population, thus decreasing FFB yield (Jackson et al. 2010).

### Impact of rainfall on oil palm production

Shortage of moisture supply in oil palm plantation can cause nutrient deficiency in oil palm trees (Teh 2016; Shafiq 2017) and affects the development of flowers, resulting in an increase in abortion, low productivity, and long inflorescences lasting approximately 8–9 months (Shafiq 2017; Woittiez et al. 2017). Low rainfall for two or more months in succession will depress OER about 11 months later (Muhamad Rizal and Tsan 2008). Excessive rainfall could lead to reduction in FFB yields and delay in the harvest (Goh et al. 2002). Heavy rain also reduces pollination, which occurs 6 months before fruit maturity (Henson et al. 2008). The amount of monthly rainfall has significant impact on oil palm FFB yield, basically through sex determination, inflorescence, abortion, and pollination (Harun et al. 2010; Oettli et al. 2018). High rainfall month is followed by reduced FFB yield after a 5-month lag period (Harun et al. 2010; Haniff et al. 2016). Similarly, a low rainfall month is followed by a high FFB yield after a 5-month lag period (Harun et al. 2010). Therefore, this signifies that the quality of FFB yield is affected by the amount rainfall usually through pollination or fruits sets. Excessive rainfall could reduce the number of sunshine hours received in the plantation which could interrupt with photosynthesis processes and synthesis of carbohydrate for the dry matter production and tissue maintenance respiration (Harun et al. 2010). Similarly, rainfall variability of ± 32% lead to decrease in palm oil earning of about $ 1181 per year (Paterson et al. 2015, 2017; Paterson and Lima 2018).

### Impact of flood on oil palm production

The 1926 storm forest flood destroyed thousands of hectares of forest and plantation in Malaysia (Koon and Kun 2006; Sani et al. 2014). Flooding turned the oil palm fibre (OPF) yellowish due to nitrogen and sulphur deficiency, which resulted in the death of immature palms (Henson et al. 2008). Flooding in a plantation is caused by two factors: high levels of water in rivers outside the plantation and heavy precipitation inside the plantation (Lohani et al. 2004; Koon and Kun 2006; Sumarga et al. 2016; Hardanto et al. 2017). A lengthy flood in a plantation will negatively affect the yield of oil palm (Simbiwen 2016). Muhadi et al. (2017) predicts flood damage in oil palm cultivation areas in Malaysia using the Average Recurrence Interval (ARI) 10 ARI, 50 ARI and 100 ARI scenarios.

Oil palm has a low tolerance for floods, which is expected to worsen as climate change continues (Hooijer et al. 2015). More so, 42% of current oil palm plantations in Sarawak, as well as many oil palms growing areas, will face increased flood problems and decreases in plantation drainage by 56% in 2059 and 82% by 2109 (Hooijer et al. 2015; Hooijer and Vernimmen 2016; Giesen and Nirmal, 2018). Projections for oil palm areas frequently flooded with high water levels, indicates decreased in planation drainability to 18% by 2009, (27%) 2034, (39%) 2059, and (64%) 2109 (Hooijer and Vernimmen 2016; Simbiwen 2016). Plantations will lose productivity as groundwater table levels fall, and floods will become more common in Sarawak, as predicted (Hooijer et al. 2015). The expectation is production of oil palm will have already been long lost prior to near-permanent floods (Hooijer et al. 2015; Hooijer and Vernimmen, 2016; Simbiwen 2016). A huge majority of plantations in Sarawak will gradually become non-productive, in decades for a large portion and within the next 100 years to encompass most of the current cultivated areas (Hooijer et al. 2015; Hooijer and Vernimmen 2016). Lowland floods in the east coast states of Kelantan, Terengganu, and Pahang affected approximately 1.02 million hectares of oil palm plantations in 2014 (United States Department of Agriculture 2015; The Star 2021). Official Malaysian government estimates recorded 230,000 tonnes decline in total production of oil palm in the Peninsular Malaysia in 2013, while production for the whole country decreased by half a million tonnes in 2014 due to floods (USDA 2015). It is estimated that yields dropped by 15% to 20% in 2021 for Sarawak (The Star 2021).

### Impact of El Niño on oil palm production

El Niño brought about extensive warming in the Eastern, the Central Pacific, and the entire Equatorial Pacific at an interval of 2–7 years (Fredriksen et al. 2020). The Malaysian climate seemed to be solely affected by El Niño, global climatic variability, and tropical monsoons controlled by both the South China Sea and the Tropical Pacific (Tangang et al. 2012). This climatic variability has significant impact on oil palm cultivation in Malaysia (Shanmuganathan and Narayanan 2012; Omar and Kamil 2017). Oettli et al. (2018) observed that when El Niño occurs in the Pacific Ocean, rainfall in Malaysia reduces but air temperature increases, generating a high level of water stress for palm trees. As a result, the yearly production of FFB becomes lower than that of a normal year since the water stress during the southwest monsoon has a significant impact on the total annual yields of FFB (Verheye 2010; Shanmuganathan and Narayanan 2012; Kamil and Omar 2017). Rising temperature condition during El Niño could result in less rainfall and increasing water stress effect to the palms (Ahmed et al. 2021). This could reduce future yields of FFB and has significant impact on CPO production (Ayat et al. 2013). CPO production fell to 8.3% in 1998, which was primarily caused by lower FFB yield harvest as a result of El Niño (Bank Negara Malaysia 1998; Hanafi et al. 2018; Sum 2018; Khan et al. 2020). The impact of water stress led to the declined of oil palm yield/ha by 16.8% during 1997/98 El Niño episode (Ariffin et al. 2003; Sum 2018). Thus, El Niño affects the processes at earlier growth stages of palms such as frond production, extent of floral abortion, the degree of survival of flowers after anthesis, and bunch weight (Verheye 2010; Kamil and Omar 2017). Malaysian CPO production in 2009 declined to 17.56 million tonnes from 17.73 million tonnes in 2008 (Abdullah 2011; Sum 2018; Nambiappan et al. 2018). This was due to the stress to the palms during the 2007/2008 El Niño episode (Abdullah 2011).

However, the biggest declined in Malaysia CPO output was reported in 1982/83 and 1997/98 were the CPO output declined by 14% and 8.3% respectively (Ivy-Ng 2015; Sum 2018). A significant decrease in oil palm yield was observed in 1983, another strong El Niño year, from 17,750kg/ha in 1981 to 20,140kg/ha in 1982, then to 15,900kg/ha in 1983, about 21.1% drop from 1982 to 1983, and then to 18,190kg/ha in 1984 (Ivy-Ng 2015; Sum 2018; Nambiappan et al. 2018). Similarly, the 1997/98 El Niño event had a significant impact on Malaysian oil palm yield and productivity (Haniff et al. 2016). Oil palm yield per hectare decreased significantly in 1998 compared to previous years and subsequent years, from 18,950kg/ha in 1996 to 19,100kg/ha in 1997, then to 15,890kg/ha in 1998, and then to 19,260kg/ha in 1999 (Ivy-Ng 2015; Sum 2018; Nambiappan et al. 2018). The 2009/2010 El Niñno event resulted in prolonged dry weather conditions in many parts of Malaysia, resulting in significantly lower palm oil supply to the world in 2010 (Hong 2015). El Niño also had an impact on oilseed production in India and Pakistan (Saini and Gulati 2014). As a result of the severe impact of 2015/2016 El Niño event palm oil production declined to 17.32 million tonnes in 2016, down from 19.96 million tonnes in 2015 (Yong 2017). Similarly, Haniff et al. (2016) observed that the FFB yield during the strong El Niño event in 2015 was slightly reduced by 0.8% to 18.48 t ha^−1^ year^−1^ from 18.63 t ha^−1^ year^−1^ in 2014. Sabah had a decline of 6.3% to 19.99 t ha^−1^ year^−1^ as compared to 21.34 t ha^−1^ year^−1^ in the previous year. This phenomenon had badly affected 7200 farmers in Peninsular Malaysia and Sabah (Abul Quasem and Gazi, 2016). The impact of El Niño differs based on its magnitude and severity (Nambiappan et al. 2018).

### Impact of La Niña on oil palm production

As a result of La Niña in 2010/2011, about 402 oil palm estates covering 428,912 ha of land were affected by inundation causing water logging, overripen and rotten of FFB (Wen and Sidik 2011; Ayat et al. 2012; Ayat et al. 2013). Consequently, the FFB losses in oil palm estates during La Niña in 2010 totalled RM 180.9 million, and RM 194.7 million in 2011 (Ayat et al. 2013). As a result of the effect of La Niña, potential income losses were estimated to be RM 155.10 million and RM 168.22 million for 2010 and 2011 period (Ayat et al. 2013). The damage to infield roads during the La Niña event was one of the reasons for the increase in the cost of FFB production in 2010 and 2011 (Ayat et al. 2012; Ayat et al. 2013). As a result, estates affected by the impacts of La Niña spent RM 25.80 million and RM 26.48 million in 2010 and 2011, respectively, to repair the roads (Ayat et al. 2012). The impact of La Niña can cause death on young palms as well as reduce yield of the older palms and disrupt many physiological processes; the rate of nutrient uptake, respiration, and photosynthesis were both depressed and this affect the growth and yield of oil palm (Corley and Tinker 2003; Henson et al. 2008; Kamil and Omar 2017). During La Niña, the transport of FFB from the estate to the oil palm mill could be disrupted (Ayat et al. 2012; Kamil and Omar 2017). La Ninña creates favourable conditions for palm trees to produce more FFB by lowering the likelihood of water stress (Oettli et al. 2018). The La Niña events right after El Niño brought about a bumper harvest and supplemented the moisture lost during the El Niño years (Tangang et al. 2017). However, subsequent year after 2015/2016 El Niño, Peninsular, and Sarawak recorded increases in FFB yield, where Peninsular improved by 3.0% to 18.77 t ha^−1^ year^−1^, while Sarawak increased slightly by 0.5% to 16.21 t ha^−1^ year^−1^ (Haniff et al. 2016)

### Impact of drought on oil palm production

Stress brought on by drought causes a decrease in biomass accumulation in oil palm plantations (Silva et al. 2017). Oil palm trees which are under drought stress are also retarded in their vegetative growth; drought stress severity and magnitude causes greatly differing responses in oil palm (Sun et al. 2011; Me´ndez et al. 2012; Azzeme et al. 2016; Silva et al. 2017). Regions that experience moderate (water deficiency of between 100 and 350 mm annually) or severe (350 mm and higher deficiency) seasons of drought every year can find their oil palm yields to be greatly stifled; the limited expansion of Amazonian oil palm plantations is actually believed to be caused by drought, among other environmental factors (Bastos et al. 2001). The production of FFB and CPO in Malaysia has been significantly reduced by droughts (12% and 10% respectively) (Adnan 2015; Amirul et al. 2016). Prolonged droughts has been reported as responsible for reducing the growth and development of oil palm, which results in lower yield (Noor and Harun 2004; Silva et al. 2016; Culman et al. 2019) and has a direct effect on FFB formation (Putra et al. 2015; Najihah et al. 2019). Plant physiology is hugely affected by drought stress, leading to the decline in productivity (Fathi and Tari 2016; Fahad et al. 2017; Kapoor et al. 2020). Changes in the morpho-anatomical, physiological, and biochemical aspects of the oil palm tree are brought on by drought stress, with these changes being measures taken by the plant to reduce water loss by transpiration and increase its water use efficiency (Kapoor et al. 2020).

### Impact of pests and diseases on oil palm production

Oil palm is susceptible to a variety of diseases, which pose a significant threat to the long-term viability of the industry (Corley and Tinker 2016). The nursing and production of oil palm tree is challenged by the impacts of pests and diseases at early and late ages of production, thus resulting in severe economic loss (Pornsuriya et al. 2013; Maluin et al. 2020). Climate change exacerbates the impacts of diseases and disease-causing organisms in oil palm plantations (Ahmed et al. 2021). Ganoderma butt rot disease (BSR) due to *Ganoderma bioninense* has been devastating over the past 100 years, particularly in Malaysia and Indonesia (Idris et al. 2004; Susanto et al. 2005; Paterson 2019; Paterson 2020a). The basal rot stem disease restricts the supply of water and essential nutrients by attacking the internal tissues and damaging the cells (Pornsuriya et al. 2013). In Malaysia, Sarawak and Sabah, the current incidences of BSR were 28%, 9%, and 23%, respectively (Idris et al. 2004; Flood et al. 2000). Kalimantan and the Philippines, Thailand, Myanmar, and Papua New Guinea would be slightly, highly, and moderately affected by BSR by 2050 (Paterson 2019; Paterson, 2020b; Lisnawita and Tantawi 2016; Merciere et al. 2017; Paterson and Lima 2018; Paterson and Lima 2012; Paterson et al. 2013).

The brown germs and seed rot due to *S. commune* was an unbearable sight in Malaysia (Dikin et al. 2003). These diseases delayed germination up to 65% and caused retarded growth (Dikin et al. 2003; Dikin et al. 2006; Arbaain et al. 2019). Other fungal diseases include *Penicillium* spp*.*, *Aspergillus* spp*.*, *F. Solani*, *Colletotrichum gloeosporioides*, and *Fusarium monoliforme* (Flood et al. 2010; Pornsuriya et al. 2013). Other distinguished diseases include *Anthrocnose* due to *Colletotrichum* that causes brown spots on the foliage and retarded growth (Flood et al. 2010; Pornsuriya et al. 2013). *Cercospora* leaf spot due to *Cercospora elaeidis* causes brownish-grey and brittle leaves that affect photosynthesis and transpiration, while *Curvularia* leaf blight due to *Curvularia orayzae* strikes during nursery stage, damages the foliage, and eventually, the whole oil palm (Flood et al. 2010; Pornsuriya et al. 2013). The *Pestalotiopsis* leaf blight causes the appearance of orange-red shade on the surface of the leaves, whereas *Cephaleuras* spp. attacks the foliage by turning it into rust colour to brown orange shade (Pornsuriya et al. 2013). The *Thielaviopsis paradoxa*, which attacks non-lignified tissue, causes the fungal disease fatal yellowing or lethal bud rot of the oil palm, providing a useful contrast to Ganoderma, whereas *Ceratocystis paradoxa* causes the disease dry basal rot (Paterson 2007; Paterson et al. 2009; Paterson and Lima, 2012). The oomycete *Phytophthora palmivora* posed a severe, low, and significant threat to Colombia and Ecuador, as well as Brazil, Malaysia, and Indonesia (Paterson, 2020a, b). Similarly, P. *palmivora* wreaking havoc on Latin American countries, particularly Colombia (Corley and Tinker 2015), destroying over 30,000 ha of oil palm plantation (Corley and Tinker 2015). The *Fusarium* vascular wilt, caused by *Fusarium oxysporum f.* sp. *elaeidis*, is the most lethal oil palm disease in Africa and South America, causing damage to oil palm plantation (Flood 2006; Cooper 2011; Paterson and Lima, 2012; Suwandi Akino and Kondo 2012). Fusarium causes two types of symptoms: acute wilt, which causes the palm to die within a few weeks, and chronic wilt, which causes the palm to live for years while stunting (Paterson and Lima, 2012). Malaysian oil palm was susceptible to infection by African Foe strains (Cooper 2011; Rusli et al. 2015). The reported insect pests included termites (Isoptera Rhinotermitidae), rhinoceros beetle (Coleoptera: Scarabaeidae), and bugworms (*Lepidoptera: Psychidae*) (Kamarudin et al. 2019). The rhinoceros beetle (Oryctes rhinoceros) is one of the most devastating pest diseases to oil palm in Malaysia (Manjeri 2014).

## Oil palm adaptation and mitigation to climate change

In order to respond to climate change, multiple efforts are sought from the society, political, economic, educational, environmental, technological, and social segments (Lahsen et al. 2010). The vulnerable areas needed adaptation and planning to ameliorate the effects of climate change include water, coastal, and marine resources; agriculture; biodiversity; forestry; as well as public health and energy (Alam et al. 2012; Tang 2019).

### Mitigation strategies to climate change

The present effects of climate change on oil palm, which have incurred losses among farmers, require mitigation measures to ameliorate such impacts and to attain sustainable production (Zainal et al. 2012; Paterson and Lima 2018; Sarkar et al. 2020; Ahmed et al. 2021). In 2009, the Malaysian government had introduced a national policy on climate change, which was designed to adapt and mitigate the effects of climate change (MNRE 2015; Rao and Mustapa 2021). These is expected to be achieved through sustainable use of natural resources and conservation of the natural environment to pursue sustainable green growth projected via Eleventh Malaysia Plan (2016–2020) by placing emphasis on key economic sectors (Economic Planning Unit 2015). Control of GHGs, especially methane through sustainable milling process and biogas capture, can protect the agro-food industry and initiate certification scheme for sustainable agricultural practices (Murad et al. 2010; Economic Planning Unit 2015; Wahid et al. 2006; MNRE 2015; Tang 2019). The mitigation measures include conservation of carbon pool, effective management of tropical forest biodiversity and carbon stored in soil, sustainable agricultural and soil management practices, zero tillage to minimise carbon loss from soil and fauna, minimal encroachment rate, and preserving dense carbon forest reserve areas and ecosystems (Smith et al. 2014; Paterson and Lima 2018; Sarkar et al. 2020). Other mitigation strategies include enacting strict regulations on indiscriminate cutting down of trees, as well as promotion of afforestation to facilitate soil conservation and ground biomass cover to enhance carbon sequestration (Burney et al. 2010; Bennetzen et al. 2012; Smith et al. 2014; Paterson and Lima 2018; Raihan et al. 2019; Sarkar et al. 2020). Another alternative pointed out refers to intensification of crop production via organic and smart agriculture (Tilman et al. 2009; Garnett et al. 2013; Smith 2013).

### Adaptation to climate change

The government (at all level) and the Non-Governmental Organizations (via extension agencies) should disseminate hands-on information to farmers (oil palm), besides creating awareness about new plantation management techniques, as well as sustainable pests and diseases control measures (Abazue et al. 2015; Ni et al. 2016). Strengthening knowledge, skills, and capacity of extension service agents facilitates in adaptation to climate change (Zikhali et al. 2020; Antwi-Agyei and Stringer 2021). More studies should look into oil palm versus climate change impacts, as well as adaptation to explore new techniques of farming practices (Wahid et al. 2006; Berry et al. 2010; Lauzon 2013; Wan Noranida et al. 2015; Hassen et al. 2016; Irawan and Syakir 2019; Man et al. 2019). Researchers are called to find ways for improving oil palm variety that is tolerant to the changing climate, new techniques of water saving to decrease infiltration, and effective measures to curb infestation of pests and diseases (Wan Noranida et al. 2015; Irawan and Syakir 2019).

Soil and water conservation management in plantation is a promising technique in adaptation to climate change (Delgado et al. 2013; Mohsen et al. 2014). Silt pit perpendicular to slope collects runoff water and circulates it within the plantation to enhance water infiltration and to decrease runoff (Lei et al. 2020). Surface runoff could be reduced by 79.41–99.0% and 71.49–74.36% using silt pit techniques and bench terrace mechanism, respectively. The two mechanisms could improve soil water retention capacity by 134.0–141.25 mm and 165.11–201.0 mm, accordingly (Murtilaksono et al. 2011; Bohluli et al. 2014, 2015).

Intercropping, such as legumes and cereals, in oil palm plantation between rows can increase the income level of farmers and improve food security, should in case of climatic uncertainties (Nchanji et al. 2016; Khasanah et al. 2020). Mulching is an effective strategy that conserves soil moisture in oil palm plantation (Mohsen et al. 2014; Iqbal et al. 2020). Mulching enhances soil structure and water holding capacity (Jordan et al. 2011; Iqbal et al. 2020; Ngangom et al. 2020; Amare and Desta 2021). Several agroecological and environment-friendly practices have been deployed in oil palm plantation to conserve soil, to minimise evapotranspiration, and to improve soil fertility (Nabara and Man 2018; Ahmed et al. 2021). Such practices include application of sustainable and environment-friendly fertiliser, monitoring of OPF size, and a highly effective management team (Murtilaksono et al. 2011; Ahmed et al. 2021). It was reported that the recommended fronds for young and matured oil palm trees are 48–56 and 40–48 fronds, respectively (Murtilaksono et al. 2011; Nabara and Man 2018). In order to minimise evaporation, pruned OPFs can be spread on the surface so as to improve soil fertility upon decay (Sutarta et al. 2011). Use of oil palm by-products, such as OPF and EFB, refers to promising soil and water conservation strategy in oil palm plantation (Teh 2016). Apart from generating massive biomass waste, 96% of oil palm above ground dry matter is recycled into various plantations which when decay and decompose to improve soil fertility, as well as to use as material for mulching (Samedani et al. 2014; Nabara and Man 2018; Ojemade et al. 2019).

Sustainable adaptation planning should be all encompassing after considering the rapid technological changes and future farming technology, which might influence adaptation options (Stringer et al. 2020; Sarkar et al. 2020). These adaptation strategies should weigh in the following factors: land tenure and fragmentation, diverse technological options, food security and sustainable ecosystem management (Iglesias and Garrote, 2018; Amjath-Babu et al. 2018), sustainable water management, capacity building, and livelihood diversification, so as to aid adaptive capacity (Naqvi et al. 2020). Realising these strategies demands political will and commitments: revisiting and reviving water, agricultural, and market policies; research and development; training for extension workers and farmers; subsidises credit to farmers; and continuous market supply despite climate change (Gruda et al. 2019; Paterson and Lima 2018; Sarkar et al. 2020).

## Conclusion

Climate change is indeed an emerging serious ecological threat. The results from this review signify that the variability of climate had, has, and will adversely affect the oil palm production. As observed, the 1997/98 and 2015/16 El Niño events had substantially reduced the yield turnover of oil palm by affecting the soil and the plant physiology merely by modifying the general weather pattern. Increase in average surface temperature displayed profound impacts on oil palm production, wherein increment in temperature by 1–4°C projected a reduction of 10–41% of oil palm yield in Malaysia. Higher temperatures exacerbate the rate of evapotranspiration, thus leaving the soil dry and aggravating water stress—ultimately resulting in retarded growth and poor harvest. Apart from increasing warmer days and colder nights, rising temperature changes the ecology of various pests and diseases. As a result, these pests and diseases become more adaptable to the changing environment and increase in population, which may create an epidemic or even a pandemic outbreak in the plantations. Pollinating insects might be susceptible to attack by other organisms, which could lead to reduction in their population, and hence, impeding the pollination process. Rainfall projection indicated torrential rain around years 2081–2100, along with wider spatial distribution and expected drier conditions during the southwest monsoon towards the end of the twenty-first century. Meanwhile, sea level has been anticipated to raise by 0.71m in 2100, in which the resulting effect is devastating flooding that could annihilate thousands of hectares of oil palm plantation, particularly those situated along the coastal zones. The study suggest some policy recommendations to address the impacts of climate change on oil palm production: sustainable land use policy and expansion without deforestation, the use of improve variety, enhance institutional research, collaboration between producer and major consumer countries in research, improve easy access, communication and availability of academic findings, technology transfer, environmental education, management skills, and extension services to oil palm growers.

## Data Availability

All data generated or analysed during the study are included in the published article(s) cited within the text and acknowledged in the reference section.
